# Antecedents of teachers’ emotions in the classroom: an intraindividual approach

**DOI:** 10.3389/fpsyg.2015.00635

**Published:** 2015-05-19

**Authors:** Eva S. Becker, Melanie M. Keller, Thomas Goetz, Anne C. Frenzel, Jamie L. Taxer

**Affiliations:** ^1^Department of Empirical Educational Research, University of Konstanz, Konstanz, Germany; ^2^Department of Empirical Educational Research, Thurgau University of Teacher Education, Kreuzlingen, Switzerland; ^3^Department of Psychology, University of Munich, Munich, Germany

**Keywords:** teacher emotions, cognitive appraisals, classroom conditions, emotional states, intraindividual approach, diary, anger, enjoyment

## Abstract

Using a preexisting, but as yet empirically untested theoretical model, the present study investigated antecedents of teachers’ emotions in the classroom. More specifically, the relationships between students’ motivation and discipline and teachers’ enjoyment and anger were explored, as well as if these relationships are mediated by teachers’ subjective appraisals (goal conduciveness and coping potential). The study employed an intraindividual approach by collecting data through a diary. The sample consisted of 39 teachers who each participated with one of their 9th or 10th grade mathematics classes (*N* = 758 students). Both teachers and students filled out diaries for 2–3 weeks pertaining to 8.10 lessons on average (*N* = 316 lessons). Multilevel structural equation modeling revealed that students’ motivation and discipline explained 24% of variance in teachers’ enjoyment and 26% of variance in teachers’ anger. In line with theoretical assumptions, after introducing teachers’ subjective appraisals as a mediating mechanism into the model, the explained variance systematically increased to 65 and 61%, for teachers’ enjoyment and anger respectively. The effects of students’ motivation and discipline level on teachers’ emotions were partially mediated by teachers’ appraisals of goal conduciveness and coping potential. The findings imply that since teachers’ emotions depend to a large extent on subjective evaluations of a situation, teachers should be able to directly modify their emotional experiences during a lesson through cognitive reappraisals.

## Introduction

“*In class I usually get angry, when I feel that my students haven’t studied enough*”

(Teacher No. 20 in a note after the diary phase)

Teachers’ emotions are an essential part of instructional settings (e.g., Hargreaves, 1998) and are related to a variety of important outcomes, such as teachers’ well-being and health (e.g., Chang, 2009; Keller et al., 2014a), classroom effectiveness (e.g., Sutton, 2005), students’ emotions and motivation (e.g., Bakker, 2005; Frenzel et al., 2009a; Radel et al., 2010; Becker et al., 2014) as well as students’ learning and performance (Pekrun et al., 2002; Beilock et al., 2010). To foster positive affective experiences in teachers, it is important to study the antecedents of their emotions. However, there is a notable lack of empirical studies focusing on teachers’ emotions and hence, only little is known about what really drives teachers’ emotional experiences in the classroom.

The present study provides the first empirical examination of key assumptions purported in Frenzel et al. (2009b) theoretical model on the antecedents and effects of teacher emotions. Students’ behaviors during a lesson (motivation and discipline) and teachers’ subjective evaluations of those behaviors and whether they align with their classroom goals (i.e., appraisals) were examined as antecedents of teachers’ experiences of enjoyment and anger. The relationships between these variables were studied on a within-person level in order to test intraindividual functioning. Thereby, a diary method with multiple measures within one teacher was employed and multilevel analyses were used to explore the antecedents of teachers’ emotions during one lesson. Furthermore, to enhance ecological validity, two sources of data were combined: teachers’ diary reports were used for the assessment of teachers’ appraisals and emotional experiences and students’ diary reports from the corresponding lessons were aggregated and used as a proxy for objective classroom conditions concerning students’ behaviors.

### Prevalence of Teachers’ Emotions

Research has shown that teachers experience a variety of emotions such as enjoyment (Sutton and Wheatley, 2003; Frenzel et al., 2009a), pride (Darby, 2008; Sutton and Harper, 2009), anger and frustration (Sutton, 2007; Chang, 2009), guilt (Hargreaves and Tucker, 1991), and anxiety (Beilock et al., 2010; Keller et al., 2014a) while being in the classroom. Most studies are based on qualitative data—however, the few quantitative studies suggest that enjoyment is the most prominent positive emotion and anger is the most frequently experienced negative emotion teachers experience while teaching (see Frenzel, 2014). Consequently, the aim of the present study was to investigate these two emotions and their possible antecedents in the classroom.

### Antecedents of Teachers’ Emotions—A Theoretical Model

Frenzel et al. (2009b; see also Frenzel and Stephens, 2013; Frenzel, 2014; Keller et al., 2014b) developed a model on the antecedents and effects of teachers’ emotions. The model is grounded in appraisal-theoretical thinking (e.g., Roseman and Smith, 2001), which states that it is not the situation itself that triggers an emotional experience, but rather an individual’s subjective interpretation of the situation (evaluative judgments; i.e., appraisals). According to Frenzel et al.’s (2009b) model, teachers’ emotions are elicited by appraisals, which depend upon teachers’ evaluations of how students’ behaviors (objective classroom conditions) relate to their goals for students’ behaviors. The model further assumes that teachers’ emotions then influence teachers’ instructional behaviors in class (e.g., motivational support and cognitive stimulation) which then impact student outcomes and behaviors (which are again an antecedent of teachers’ emotions). The present study focused only on examining the antecedents of teachers’ emotions and did not investigate the effects or the reciprocal relations proposed by the model. Specifically, the present study focused on empirically examining student behaviors and teacher appraisals as antecedents of teacher emotions.

#### Student Behavior as an Antecedent of Teacher Emotions

Frenzel et al.’s (2009b) model assumes that students’ classroom behaviors have an impact on teachers’ emotional experiences. This claim is also supported by various empirical results. For example, previous studies have shown that high achieving and highly motivated students are a source of positive emotional experiences for teachers (e.g., Hargreaves, 2000; Zembylas, 2002; Frenzel and Götz, 2007; Frenzel et al., 2009b). In particular, research has shown that students’ motivational engagement is related to teachers’ emotions: independent of students’ cognitive abilities and performance, teachers prefer to teach students who work hard and invest effort (Covington and Omelich, 1979; Biddle and Goudas, 1997). Students’ misbehavior has been shown to be a key eliciting factor of negative emotions in teachers (Brophy and McCaslin, 1992; Sutton and Wheatley, 2003; Frenzel and Götz, 2007). Students who disrupt a lesson and do not follow the classroom rules adversely affect teachers’ classroom instruction and performance, jeopardize teachers achieving their classroom goals, and such misbehavior can have long-term effects on teachers’ well-being and emotional exhaustion (Ben-Ari et al., 2003; Tsouloupas et al., 2010; Spilt et al., 2011; Chang, 2013). However, the cited empirical results are mostly based on qualitative studies and the few quantitative studies relied on teachers’ perceptions of students’ behaviors. Assessing data via self-reports from only one source (i.e., the teacher) can yield inflated correlations and can be one of the main sources of measurement error (e.g., Podsakoff et al., 2003). To the authors’ knowledge, there are no empirical results pertaining to teachers’ emotions as related to students’ actual behaviors (i.e., student-reported). Therefore, to enhance ecological validity, the current study focused on classroom conditions as seen through the students’ rather than the teachers’ eyes. This was accomplished by using averaged student-reports from one lesson on their motivation and discipline levels and investigating their relevance for teachers’ appraisals and emotional experiences in the corresponding lessons.

#### Teachers’ Appraisals Mediating the Relationship Between Student Behaviors and Teacher Emotions

A key assumption in Frenzel et al.’s (2009b) model is that the relationship between students’ behaviors and teacher’s emotions is mediated by appraisals. At certain points during or shortly after a lesson, teachers appraise students’ behaviors in accordance with their goals for that particular lesson. Based on the most commonly agreed upon appraisals in the literature (see, e.g., Ellsworth and Scherer, 2003; Zembylas, 2004), central appraisals for teachers’ emotions in Frenzel et al.’s (2009b) theoretical model are goal conduciveness, goal importance, accountability, and coping potential.

The appraisal of *(un)conduciveness* comes first in the appraisal process (e.g., Scherer, 2001) and determines the valence of a teacher’s emotional reaction. If an event is appraised as harmful or threatening to one’s goals, the resulting emotion will be negative, but if the event is appraised as beneficial, it will be positive. The intensity of the resulting emotion is then determined by the *importance of the goal*; the more important the goal, the more intense the occurring emotion will be. When there is no goal at stake, no emotion will emerge, with the possible exception of boredom (Pekrun et al., 2010). *Accountability* appraisals refer to the perceived responsibility for an event or action, that is, whether it is oneself—or someone else—who is perceived as being responsible. *Coping potential* refers to appraisals about the strength of one’s personal control over events and actions. Both accountability appraisals and coping potential determine the valence and intensity of the emotion. Frenzel et al. (2009b) and Frenzel (2014) proposed each of these appraisal dimensions as important for the formation of teachers’ emotions; however, empirical support for this supposition is still lacking.

The present study aimed to empirically investigate the link between classroom conditions, teachers’ appraisals and teachers’ emotions and focused on the appraisals of *goal conduciveness* and *coping potential* as these appraisals are important in all classroom situations. Appraisals of *goal importance* require establishing the importance of classroom goals before a lesson starts (e.g., is it very important for the teacher to maintain discipline during the upcoming lesson?), whereas *accountability* appraisals require specifying a particular event and classifying it as being goal-conducive or unconducive (e.g., an intense classroom disruption or a particularly engaged student). Therefore, our diary approach assessing teachers’ emotional experiences and appraisals while judging the entire lesson retrospectively was only suitable for testing the two appraisals of goal conduciveness and coping potential.

### Intraindividual Approaches to Study Teachers’ Emotions

Similar to general appraisal theories of emotion, some of the assumptions in Frenzel et al.’s (2009b) model of teachers’ emotions are based on situation-specific considerations: a momentary event, such as students’ misbehavior, is perceived and appraised accordingly and this appraisal then leads to a corresponding emotional response. Therefore, the situation–appraisals–emotion link occurs across situations and within teachers. These situation-specific assumptions of the theoretical model of teachers’ emotions should be tested with corresponding intraindividual analyses. Yet to date, most studies on teachers’ emotions have focused on trait-reports (habitual experiences), and investigated interindividual relations or they used interindividual analyses to test intraindividual functioning (for a critique, see, e.g., Pekrun and Schutz, 2007; Ahmed et al., 2010). However, inter and intraindividual analyses are statistically independent, and it is essentially not possible to draw conclusions for intraindividual relations from interindividual data, and vice versa (e.g., Molenaar, 2004; Johnston and Johnston, 2013; Adolf et al., 2014; Voelkle et al., 2014).

In a response to this critique, intraindividual, real-life approaches (i.e., experience sampling studies) have been employed to study the role of appraisals for students’ emotions (Ahmed et al., 2010; Bieg et al., 2013), but to date there are only isolated studies that used an intraindividual approach to measure teachers’ emotions (diary approach: Frenzel and Götz, 2007; experience-sampling approaches: Carson et al., 2010; Keller et al., 2014a) and none of them addressed appraisal-emotion links.

Experience sampling approaches are an important method in emotion research as they minimize retrospective biases such as retrieval distortions, cognitive and memory limitations, or influences from personality factors or social desirability (e.g., Barrett, 1997; Carson et al., 2010). However, as the present study focused on a variety of antecedents of teacher emotions (i.e., situational characteristics as reported by the whole class and different teacher appraisals), a great deal of information needed to be obtained. An experience sampling design with random signals during a lesson (as employed in the study by Keller et al., 2014a) would have required the teachers and their classes to actually interrupt the lesson to fill out the experience sampling-questionnaires, and thus would have been too invasive. Therefore, the present study used a diary approach in order to obtain information on situational characteristics, appraisals and emotions without disrupting instructional processes and still keeping retrospective biases to a minimum. A further advantage of using an intraindividual diary approach is the fact that it is capable of capturing the dynamics of emotions (i.e., temporal variations) and their antecedents in the classroom. Previous research on academic emotions suggests that there is a considerable amount of intraindividual variability in emotional experiences across and within subject domains (e.g.,Goetz et al., 2010, 2013; Nett and Goetz, 2011; Bieg et al., 2013) and that appraisals also vary on a day-to-day level and can be considered as a context sensitive construct (Boekaerts, 2001; Ahmed et al., 2010). The present study, therefore, also explores the amount of within-person variability for emotional experiences and their antecedents.

## Research Hypotheses

**FIGURE 1:**
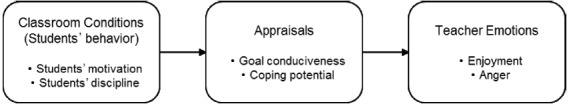
**Figural representation of the present study’s key assumptions based on Frenzel et al.’s (2009b) model on the antecedents of teachers’ emotions**.

In responding to a notable lack of research addressing the antecedents of teachers’ emotions in the classroom, the present study tested assumptions of Frenzel et al.’s (2009b) model of teachers’ emotions which posits classroom conditions as central antecedents of teachers’ emotions via teachers’ appraisals (see Figure 1). The objectives of the study were firstly to examine the relationship between classroom conditions (students’ motivation and discipline) and teachers’ experiences of enjoyment and anger; and secondly, to examine the mediating role of teachers’ appraisals in the relationship between classroom conditions and teachers’ emotions.

### Hypothesis 1

Classroom conditions (students’ reports on motivation and discipline levels) are related to teachers’ emotional experiences during the same lesson. Specifically, students’ motivation and discipline are positively related to teachers’ enjoyment (H1a) and negatively related to teachers’ anger (H1b).

### Hypothesis 2

Appraisals mediate the relationship between classroom conditions and teachers’ emotions. Appraisals of goal conduciveness and coping potential positively influence teachers’ enjoyment and negatively influence teachers’ anger.

## Materials and Methods

### Ethical Statement

The present study was conducted in compliance with ethical standards provided by the Federation of German Psychologists Association (Berufsverband deutscher Psychologinnen und Psychologen, 2005) and the American Psychological Association (2010). Guidelines provided by these institutions state that formal informed consent is not obligatory when no potential harm or distress is to be expected and/or when normal educational practices are followed as a goal of the research. Prior to participation, teachers and students were informed about the goals of the research, duration, procedure and anonymity of their data. Participation was voluntarily and it was possible to withdraw participation at any time. Verbal informed consent prior to data collection was provided by all teachers and students. Data was collected and analyzed anonymously; all identifiers that could link individual participants to their results were removed and destroyed after data entry.

### Sample and Procedure

For the present study, 39 secondary school mathematics teachers from the highest track of the German school system (i.e., Gymnasium; about one-third of a student cohort attend this school track; Federal Statistical Office, 2014) participated together with one of their 9th or 10th grade classes (N = 758 students). Teachers were on average 39.53 years old (SD = 11.40 years) and 49% of them were female. Students were on average 15.60 years old (SD = 0.72) and 55% of them were female. Diary data was collected in a total of N = 316 lessons, which resulted in an average of 8.10 lessons per teacher and class.

Trained research personnel gave teachers and students a diary (a small booklet consisting of the state-level questionnaires) and briefly instructed them on how to fill it out. The diary was designed to sample 5–10 lessons per class, thus lasting 2–3 weeks in which the teacher and the whole class filled in the short state-questionnaire in the last 5 min of each mathematics lesson. The teachers initiated the diary data collection in their classrooms without any trained research personnel present. Teachers were requested to end their lessons 5 min early so that their students and they could fill out the diaries. To ensure that teachers did not have access to their students’ diaries, students were responsible for their own diaries and brought it with them to each mathematics lesson. Furthermore, students and teachers used an individual code instead of their names in the diary so that the data remained anonymous. After the last assessment, trained research personnel collected the diaries. For participating in the study, classes received 50 euros for their class fund and teachers were compensated with a book voucher.

### Measures

#### Teachers’ Self-reported Emotions

Teachers’ experiences of enjoyment and anger were assessed with two items each. Items were based on trait measures from the Achievement-Emotion Questionnaire for Teachers (Frenzel et al., 2010) as well as a previously conducted momentary assessment approach (Keller et al., 2014a). Items were adapted to suit the diary-based assessment of emotional experiences after each lesson. The item formulations were as follows: “In this lesson, I enjoyed teaching,” “In this lesson, I often thought this is going great!” for teachers’ enjoyment and “In this lesson, I often had reasons to be angry,” “In this lesson, teaching frustrated me” for teachers’ anger. Cronbach’s alpha ranged from 0.62 to 0.79 for teachers’ enjoyment and from 0.66 to 0.91 for teachers’ anger for the 10 different assessment points (teachers and students filled in the diaries in 5–10 lessons, see above). All items were rated on a scale from (1) *strongly disagree* to (5) *strongly agree*.

#### Teachers’ Self-reported Appraisals

Due to time constraints, teachers’ appraisals were assessed with single-items, which is a common practice among studies using real-life data with multiple assessments (e.g., Schimmack, 2003; Tong et al., 2007; Goetz et al., 2013; Keller et al., 2014a). Item formulations were as follows: “In this lesson, students’ behavior was beneficial for my lesson goals” for goal conduciveness and “In this lesson, I felt like I had everything under control” for coping potential. Items were rated on a 5-point Likert scale ranging from (1) *strongly disagree* to (5) *strongly agree*.

#### Student-reported Class Motivation

Students’ motivation was assessed based on the conceptualization of interest, a specific form of intrinsic motivation, which consists of two facets: positive emotional experiences and personal relevance or value (see, for example, Krapp, 2007). For the emotion-related facet, two items were selected from the Academic Emotion Questionnaire (Pekrun et al., 2005), and for the value-facet, one item from a scale employed in the PALMA project (Pekrun et al., 2007) was utilized. Items were adapted to suit the diary method and assessment after one specific lesson and formulated as follows: “This math lesson was fun for me,” “I enjoyed this lesson” (emotion-related facet), and “In this lesson, math was important to me regardless of grades” (value-related facet). Items were rated on a 5-point Likert scale ranging from (1) *strongly disagree* to (5) *strongly agree*. Cronbach’s alpha for the three items ranged from 0.69 to 0.76 for the 10 different assessment points, indicating acceptable homogeneity of the scale. Mean scores for the scale were aggregated for all students of one class onto the lesson level (i.e., one score per lesson per teacher) to obtain an indicator of class motivation during one lesson. That is, 5,271 student ratings (16.68 per lesson) were aggregated to 316 ratings (one aggregated score for each lesson). This procedure was admissible because as long as there is sufficient homogeneity among the students, aggregated student ratings have been found to be fairly objective indicators for actual classroom conditions (Lüdtke et al., 2006). The intraclass correlation [ICC(2)] gives an estimate of the reliability of the aggregated variable. ICC(2) for class motivation was 0.70, indicating that aggregated class motivation yielded adequately objective estimates of what was happening within a particular lesson.

#### Student-reported Class Discipline

Class discipline was assessed with two selected items from a scale developed for the COACTIV project (Baumert et al., 2009) referring to classroom management in the sense of few classroom disturbances and effective use of time. Item formulations were as follows: “In this lesson, instruction was often disrupted” and “In this lesson, a lot of time was wasted.” Both items were rated on a scale from (1) *strongly disagree* to (5) *strongly agree* and were reverse coded before further analyses. Cronbach’s alpha for the items ranged from 0.70 to 0.83, indicating acceptable homogeneity of the scale. Students’ individually perceived discipline was also aggregated onto the lesson level in order to obtain a proxy for class discipline during the lesson. ICC(2) for class discipline was 0.85, indicating good reliability of aggregated student reports on discipline within a particular lesson.

### Analyses

The data of the diary assessment represents a nested data structure, with teacher diary entries (i.e., lessons, *N* = 316) nested within teachers (or classes, as each teacher participated with only one class; *N* = 39). The average cluster size was 8.10, meaning that on average each teacher and their class filled out the diary in eight lessons.

In order to correctly estimate standard errors, multilevel analyses were applied, which take the nesting of lessons within teachers into account. As the study hypotheses refer only to within-person relations, all relationships were modeled on the within-level, that is, lesson level. To this end, independent variables were group mean centered to focus on relations occurring within persons. Multilevel structural equation models (MSEM) were estimated utilizing the software *Mplus* 7.0 (Muthén and Muthén, 1998–2012). Beyond chi-square statistics, the fit parameters root mean square error of approximation (RMSEA; cut-off <0.05), comparative fit index (CFI; cut-off >0.95), and standardized root mean square residual (SRMR) for the within-level (cut-off <0.05) are reported for model fit of the MSEMs (see, for example, Hu and Bentler, 1999).

## Results

### Descriptive Results

Descriptive results (means, standard deviations, percentage of within-teacher variability and within-teacher correlations) for all study variables are provided in Table 1. Means and standard deviations were obtained by averaging across all teachers/classes and measurement points. Percentage of within-teacher variability can be interpreted as the percentage of variance that lies within teachers (Level 1) and was calculated with [1 – ICC(1)] × 100. Intercorrelations of study variables are displayed as occurring within teachers (intraindividually), that is on Level 1. As such, those correlations describe the extent to which two constructs co-occur, on average, in the same lesson.

**TABLE 1:** **Descriptive statistics and intercorrelations for study variables**.

	*M*	SD	% of within-teacher variability	Intercorrelations				
				(1)	(2)	(3)	(4)	(5)
Classroom conditions								
(1) Class motivation	2.87	0.42	53					
(2) Class discipline	3.91	0.52	47	0.24*				
Appraisals								
(3) Goal conduciveness	3.95	0.90	74	0.31**	0.33**			
(4) Coping potential	4.14	0.87	69	0.26**	0.23**	0.42**		
Emotions								
(5) Enjoyment	3.76	0.85	84	0.38**	0.24**	0.58**	0.53**	
(6) Anger	1.63	0.79	79	–0.29**	–0.37**	–0.58**	–0.51**	–0.65**

All items were rated on a scale from (1) to (5). Means were calculated based on manifest variables and averaged across all lessons and teachers. Lessons (N = 316) were nested within teachers (N = 39). Percentage of within-teacher variability were calculated as follows: [1 – ICC(1)] × 100. Intercorrelations were calculated based on manifest variables and are displayed as occurring within teachers (intraindividually). *p < 0.01, **p < 0.001.

Regarding mean levels of teachers’ emotions, teachers reported relatively high mean scores for enjoyment (*M* =3.76), while anger was reported less intensely, yet still substantially (*M* =1.63). Mean scores of classroom conditions indicate that students rated their motivation and discipline level relatively highly (*M* = 2.87 and *M* = 3.91, respectively on scale ranging from 1 to 5).

Within-teacher variability for teachers’ emotions and appraisals were similar in magnitude (0.84 for enjoyment, 0.79 for anger, 0.74 for goal conduciveness, and 0.69 for coping potential), indicating that most of the variance originated from situational variation within teachers (69–84%) and only 16–31% can be attributed to between-teacher variation. In comparison, withinteacher variation for classroom conditions was considerably low (0.47 for discipline and 0.53 for motivation). Thus, variance in classroom conditions can be equally attributed to situational variation and stable characteristics of the teacher or the class.

Intercorrelations of study variables show that on average teachers’ appraisals were correlated with enjoyment and anger in the hypothesized directions. Furthermore, classroom conditions as reported by students were related to teachers’ self-reported emotional experiences in the hypothesized direction. Teacher enjoyment and anger were negatively correlated. That is, if more enjoyment was experienced within a lesson, then less anger was reported.

### Classroom Conditions Predicting Teachers’ Emotions

According to Hypothesis 1, class motivation and discipline (classroom conditions) should relate to teachers’ momentary experiences of enjoyment and anger. In order to test this assumption, two MSEMs were run, one for each teacher emotion (enjoyment and anger). Classroom conditions and teachers’ emotions were modeled as latent variables and emotions were predicted by classroom conditions only on the within level (i.e., intraindividually). The regression coefficients are shown in Table 2.

**TABLE 2:** **Teachers’ emotions predicted by classroom conditions**.

Classroom conditions	Enjoyment		Anger	
	β	SE	β	SE
Class motivation	0.37***	0.08	–0.20**	0.07
Class discipline	0.24**	0.07	–0.43***	0.08
*R*^2^	0.24		0.26	

Dependent and independent variables were all modeled as latent variables. Classroom conditions were correlated with each other. All relations were modeled only on the within level, with the indicators for independent variables being group mean centered. R^2^ refers to the explained variance on the within level. Model fit for the respective models was: enjoyment: χ^2^ = 19.36, df = 12, p = 0.08, RMSEA = 0.04, CFI = 0.99, SRMR_within_ = 0.04; anger: χ^2^ = 22.53, df = 11, p = 0.02, RMSEA = 0.06, CFI = 0.98, SRMR_within_ = 0.04. **p < 0.01, ***p < 0.001.

The analyses indicate that both teacher emotions were related to students’ reports on motivation and discipline during one lesson. Specifically and as hypothesized, high levels of class motivation and discipline within one lesson corresponded to teachers reporting higher levels of enjoyment (H1a) and lower levels of anger (H1b). Explained variances in teachers’ emotions were about equal for enjoyment and anger (24% and 26%, respectively).

### Classroom Conditions Predicting Teachers’ Emotions via Appraisals

**FIGURE 2:**
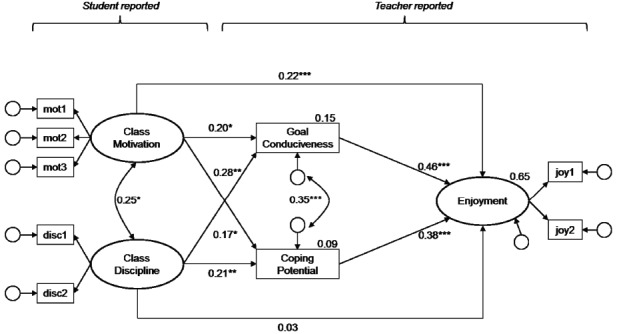
**Teacher appraisals mediating the relationship between classroom conditions and teacher enjoyment.** Standardized coefficients are shown; the regression coefficients for the latent variable indicators and residuals are not displayed. Estimates at the dependent variables represent explained within-level variance (*R*^2^). Model fit: *χ*^2^ = 36.97, *df* = 19, *p* = 0.01, RMSEA = 0.05, CFI = 0.97, SRMR_*within*_ = 0.04. *p < 0.05, **p < 0.01, ***p < 0.001.

**FIGURE 3:**
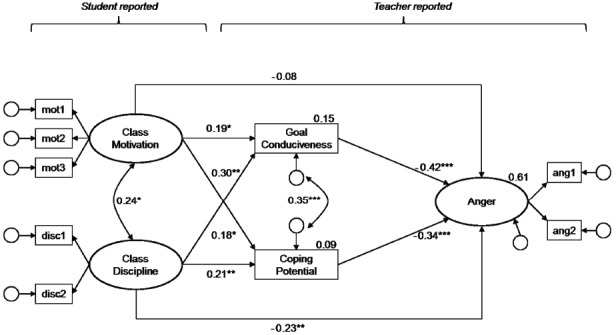
**Teacher appraisals mediating the relationship between classroom conditions and teacher anger.** Standardized coefficients are shown; the regression coefficients for the latent variable indicators and residuals are not displayed. Estimates at the dependent variables represent explained within-level variance (*R*^2^). Model fit: *χ*^2^ = 35.11, *df* = 19, *p* = 0.01, RMSEA = 0.05, CFI = 0.97, SRMR_*within*_ = 0.04. *p < 0.05, **p < 0.01, ***p < 0.001.

According to Hypothesis 2, teachers’ appraisals (goal conduciveness and coping potential) should mediate the relationship between classroom conditions and teachers’ emotions. To test this, two separate MSEMs (one for each emotion) were run with classroom conditions predicting teachers’ appraisals which in turn predict their emotions. Classroom conditions as well as teachers’ emotions were again modeled as latent variables, but appraisals were included as manifest variables because they have been assessed with single-items. The results are shown in Figures 2 and 3. Overall, both models achieved good model fit. Together, classroom conditions and teachers’ appraisals explained about two-thirds of the within-person variance in teachers’ enjoyment and anger (65 and 61%, respectively). Thus, the explained variance increased considerably as compared to the model in which only classroom conditions were considered as antecedents.

#### Classroom Conditions Predicting Teachers’ Appraisals

Students’ aggregated reports of class level motivation and discipline were related to both teachers’ perceptions of goal conduciveness and coping potential. Specifically, teachers reported higher levels of coping potential and perceived students’ behavior as being more conducive to their goals in lessons in which the class was highly motivated and disciplined.

#### Appraisals Predicting Teachers’ Emotions

Teachers’ emotions were significantly predicted by both appraisals. Specifically, the more teachers perceived their students’ behavior as conducive to their goals, and the more they reported having things under control within one lesson, the more enjoyment and less anger they reported.

#### Mediation via Teachers’ Appraisals

Overall, the effects of classroom conditions on teachers’ emotions were partially mediated by teacher appraisals. For teachers’ enjoyment, the direct effect of class motivation on enjoyment was still significant after introducing the appraisals (β_direct_ = 0.22, *p* < 0.01; total indirect effect of class motivation on enjoyment: β_indirect_ = 0.15, *p* < 0.05). The effect of class discipline was fully mediated by the appraisals with the direct effect rendered close to zero (β_direct_ = 0.03, *p* > 0.05; total indirect effect of class discipline on enjoyment: β_indirect_ = 0.21, *p* < 0.01).

For teachers’ anger, the effect of class motivation was fully mediated by teacher appraisals (β_direct_ = –0.08, *p* > 0.05; total indirect effect of class motivation on anger: β_indirect_ = –0.14, *p* < 0.05), whereas the effect of class discipline was partially mediated with the direct effect of class discipline on anger being still significant after introducing the appraisals (β_direct_ = –0.23, *p* < 0.01; total indirect effect β_indirect_ = –0.20, *p* < 0.001).

## Discussion

This research addressed a largely unexplored field in empirical educational research, namely antecedents of teachers’ emotions in the classroom. For the first time, some of the key theoretical propositions outlined in Frenzel et al.’s (2009b) model regarding the antecedents of teachers’ emotions in the classroom were tested empirically. More specifically, the present study explored the relationship between classroom conditions (as perceived by students) and teachers’ emotions and the mediating role of teachers’ subjective appraisals in this relationship. To improve the ecological validity of the findings and to allow for intraindividual analyses, a diary approach with teachers and students was employed.

### Prevalence and Intraindividual Variability of Teachers’ Emotions, Teachers’ Appraisals and Classroom Conditions

Mean levels in teachers’ experiences of enjoyment and anger corroborate previous findings that teachers predominantly experience positive emotions related to teaching (e.g., Keller et al., 2014c). This supports the assumption that interactions with students can be charged with positive emotions and offer emotional rewards (e.g., Hargreaves, 2005). In line with previous research on teachers’ emotional experiences in the classroom (Frenzel and Götz, 2007; Keller et al., 2014a), we found that the vast majority of variance in teachers’ enjoyment and anger, namely about 80%, lies within individuals. That is, each teacher’s emotions vary strongly from lesson to lesson. Similarly and in line with research concerning students’ appraisals in class (Ahmed et al., 2010), teachers’ appraisals also showed to be highly situation specific (about 70% of variance lying within teachers and across situations). In slight contrast, variance in classroom conditions was equally distributed across both levels, indicating that about half of the variance in students’ behavior lies within teachers and across situations (Level 1), and half of the variance can be attributed to differences between teachers (Level 2). Since each teacher only participated with one class, it is not possible to determine whether Level 2 variance actually pertains to stable personal characteristics between the teachers (e.g., knowledge of classroom management strategies) or to stable characteristics between the classes (e.g., class with many highly motivated students). Nevertheless, the results from the present study show that students’ behaviors regarding motivation and discipline were more stable for one teacher (or class) than teachers’ emotions or appraisals, yet they still varied considerably from lesson to lesson. This indicates that there is no such thing as classes that are always motivated and disciplined or teachers who are always capable of motivating and disciplining their classes.

### Classroom Conditions Predicting Teachers’ Emotions

The present study examined the link between student-reported classroom conditions (students’ behaviors regarding their motivation and discipline during one lesson) and teachers’ emotions during the lesson. Students’ motivation was the strongest predictor of teachers’ enjoyment. This is also in line with previous findings indicating that teachers profit on an emotional level the most when students are motivated, engaged and show personal growth (Stenlund, 1995; Frenzel and Götz, 2007; Frenzel et al., 2009b). However, these studies focused solely on *teachers’ perceptions* of student motivation rather than gauging students’ actual in-class motivation and engagement. In contrast to enjoyment, teachers’ anger was primarily related to students being undisciplined. This aligns with findings from previous studies that utilized different methodological approaches (Frenzel and Götz, 2007; Frenzel et al., 2009b; see also Sutton, 2007; Chang and Davis, 2009). However, these previous studies also relied exclusively *on teachers’ perceptions* of students’ behaviors. Thus, the present study demonstrates that classroom motivation and discipline as assessed via aggregated student ratings are important antecedents of teachers’ experiences of enjoyment and anger.

### Appraisals as Mediators

Theoretical underpinnings in appraisal theories of emotion (e.g., Ellsworth and Scherer, 2003) assume that the situation itself is not the central factor for the emergence of emotions. Rather, the subjective interpretation of situational characteristics determines emotional experiences. For teachers this means that classroom conditions, such as students’ motivation during a lesson, should not directly impact teachers’ emotions, but rather the influence of classroom conditions on teachers’ emotional experiences should be mediated through teachers’ subjective appraisals (see Frenzel et al., 2009b; Frenzel, 2014). This assumption was explored in the present study by investigating whether two important appraisals—goal conduciveness and coping potential—mediate the effect of students’ motivation and discipline level on teachers’ enjoyment and anger.

The results showed that goal conduciveness and coping potential fully mediated the effect of students’ discipline level on teachers’ experiences of enjoyment, whereas goal conduciveness and coping potential only partially mediated the effect of students’ motivation level on teachers’ experiences of enjoyment. There was still a small and positive direct effect of students’ motivation on enjoyment. This direct effect could possibly be explained by emotional contagion processes (Hatfield et al., 1993, 1994). Emotional contagion theory states that emotions can directly and unconsciously be transmitted from one individual to another through the synchronization of “facial expressions, vocalizations, postures and movements with those of another person” (Hatfield et al., 1994, p. 5). Since the present study’s measure for students’ intrinsic motivation included an affective component, students’ positive affect could be directly related to teachers’ enjoyment (as also shown by Bakker, 2005; Frenzel et al., 2009a; Becker et al., 2014).

Goal conduciveness and coping potential fully mediated the effect of students’ motivation level on teachers’ experiences of anger, whereas goal conduciveness and coping potential only partially mediated the effect of students’ discipline level on teachers experiences of anger. There was still a small direct negative effect of students’ discipline level on teachers’ anger even when accounting for teachers’ appraisals. This is not surprising, since accountability appraisals are also considered important for the emergence of teachers’ anger; however, they could not be included in the present study due to the study design. Within the present study teachers’ reported on their appraisals and emotions in all lessons, independently of whether they achieved their classroom goals. Assessing accountability appraisals would require specifying a particular event and classifying it as either goal conducive or unconducive, which is not feasible for a diary-approach.

Overall, the key assumption that appraisals (partially) mediate the effects of classroom conditions on teachers’ emotions could be supported. The study findings further highlight the relative importance of teachers’ appraisals of situations as compared to actual classroom events. Specifically, objective classroom conditions alone explained only 24 and 26% of situational variance in teachers’ enjoyment and anger, respectively. After including goal conduciveness and coping potential as two key appraisals, the amount of explained variance increased to 65 and 61% for teachers’ enjoyment and anger, respectively. Thus, not only is it important what happens in class and while teaching and interacting with students, but even more so how teachers interpret and appraise these events.

### Limitations

The present study was designed so as to overcome some limitations of previous studies. First of all, by utilizing a diary approach to assess teachers’ self-reports of appraisals and emotions, retrospective bias of emotional trait reports (see Robinson and Clore, 2002) could be kept to a minimum. Secondly, by introducing student reports as a proxy for classroom conditions, the single-source bias of earlier studies was overcome. Nevertheless, the present study has its own limitations.

Even though the present study used two data sources (teachers and students), it still relied on self-reports. Future studies could also integrate physiological measures to assess teachers’ and students’ emotions. Furthermore, external observer ratings could be used to disentangle students’ and teachers’ diary reports on what was happening within a particular lesson.

Another important limitation regards the study sample. Teachers were recruited based on voluntary participation and could personally choose—in case they had more than one 9th or 10th grade class in mathematics—with which class they wanted to participate. This could have resulted in a bias in the direction of generally highly motivated teachers and well-disciplined and highly motivated classes. Furthermore, the sample size is rather small; although 39 teachers (as the number of units on Level 2 in multilevel analyses) should yield reliable results (Maas and Hox, 2005), a validation of the present study findings with a larger sample would be desirable. This also pertains to the breadth of the sample, which included only secondary school teachers from Gymnasium and 9th or 10th grade mathematics classes. Although from a theoretical viewpoint, no differences in relations between classroom conditions, appraisals, and emotions should be expected for different school tracks, subjects, or age level of students, this needs to be corroborated in future studies.

A third limitation pertains to the chosen appraisals within the present study. As a consequence of the study design relying on diaries and evaluations of appraisals and emotions pertaining to one lesson, only the appraisals of goal conduciveness and coping potential were included. Yet, undoubtedly, other appraisals also play a role in the emergence of teacher emotions (e.g., goal importance, accountability). Future studies should use designs that allow for testing these appraisals and how they relate to teachers’ emotions.

Finally, it should be noted that the direction of influence between classroom conditions, teachers’ appraisals and teachers’ emotions is while reasonable and based on theoretical considerations, is correlational in nature. Relations, especially regarding the link between classroom conditions and teachers’ emotions (Hypothesis 1) are likely reciprocal and also assumed as such in Frenzel et al.’s (2009b) theoretical model (Frenzel et al., 2009b; Frenzel, 2014). Previous research has also investigated teachers’ emotions as the source of students’ motivation via their instructional behavior (Bakker, 2005; Frenzel et al., 2009a; Kunter et al., 2013). Future studies could focus on such reciprocal effects between students and teachers by using repeated assessments in various lessons within 1 day to model initial levels of emotions from the previous lesson. Such a design would require a sample with teachers who teach more than one subject in one class (e.g., primary school teachers), and have multiple lessons with the same class each day.

### Implications

Given that the present study investigated the impact of classroom conditions and teacher appraisals on teachers’ in-class experiences of enjoyment and anger on an intraindividual level, several important implications can be derived pertaining to optimizing teachers’ emotional experiences in class and thereby contributing to their overall health and well-being. At first glance, a seemingly trivial implication pertains to the prevalence of teachers’ enjoyment, which is in line with previous research (e.g., Frenzel and Götz, 2007; Keller et al., 2014b). Despite the fact that teachers also report to find their job exhausting (see, e.g., Keller et al., 2014a), they evidently at the same time experience their interactions with students as highly rewarding (see also Hargreaves, 2005). From a theoretical viewpoint, the experience of positive emotions can be considered a resource individuals can actively draw on and benefit from (Fredrickson and Joiner, 2002); thus, helping teachers to become aware of the presence and strength of their enjoyment in the classroom (e.g., by using emotion-diaries or enhancing mindfulness) could improve their well-being and ultimately make them more resilient in the face of pressure and stress.

A second implication regards the present study’s finding that variance in students’ motivation and discipline can be equally attributed to situational characteristics of the lesson and to stable characteristics of the teacher or class. Thus, interventions which target improving teachers’ emotional experiences through adapting classroom conditions consequently need to tackle both levels simultaneously: first, interventions need to address the high variability of student motivation and discipline across situations (i.e., from lesson to lesson). Teachers should be reminded of that fact and strive toward accepting that sometimes students are more distracted or less motivated due to situational constraints. Thus, teachers should adjust goals in a realistic way (for example, not all students need to be motivated in all lessons) so that they are protected against experiencing frustration while teaching a lesson. Second, interventions could offer for example on-the-job or video-based trainings on classroom management strategies (e.g., Webster-Stratton et al., 2011; Gaudreau et al., 2013; Gold et al., 2013) or motivation strategies (e.g., Jaakkola and Liukkonen, 2006) so that teachers can aim at strengthening students’ overall discipline and motivation levels (see also Briesch and Chafouleas, 2009).

A third highly important practical implication regards the dependency of teachers’ emotional experiences primarily on their own appraising of the situation, indicating that teachers can actively alter their own emotional experiences by adapting their interpretation and evaluation of a situation. A highly adaptive way of doing so is through cognitive reappraisal strategies (see, e.g., Gross and John, 2003), which are trainable in individuals (e.g., Garland et al., 2011; Jamieson et al., 2013; Denny and Ochsner, 2014). Futures studies could consider how such an intervention needs to be designed in order to instill effective and adaptive cognitive reappraisal strategies in teachers so that they benefit emotionally.

In conclusion, the present study gives important insights into the functioning of situational characteristics, teachers’ evaluations thereof and corresponding emotional responses. It thus, advances our understanding of the involved processes on an intraindividual level and derives not only vantage points for future in-depth studies, but also important practical implications for teachers.

### Conflict of Interest Statement

The authors declare that the research was conducted in the absence of any commercial or financial relationships that could be construed as a potential conflict of interest.
